# Lung cancer metastasis-related protein 1 promotes the transferring from advanced metastatic prostate cancer to castration-resistant prostate cancer by activating the glucocorticoid receptor α signal pathway

**DOI:** 10.1080/21655979.2021.2020397

**Published:** 2022-02-20

**Authors:** Kai Wang, Xuliang Wang, Xian Fu, Ji Sun, Liwei Zhao, Huadong He, Yi Fan

**Affiliations:** a Department of Urology, Affiliated Hangzhou First People’s Hospital, Zhejiang University School of Medicine, Hangzhou, Zhejiang Province, China; b Department of Urology, Affiliated Xiaoshan Hospital, Hangzhou Normal University, Hangzhou, Zhejiang Province, China

**Keywords:** Prostate cancer, LCMR1, GRα, androgen-dependent prostate cancer

## Abstract

Androgen deprivation therapy is currently the main therapeutic strategy for the treatment of advanced metastatic prostate cancer (ADPC). However, the tumor type in ADPC patients transforms into castration-resistant prostate cancer (CRPC) after 18–24 months of treatments, the underlying mechanism of which remains unclear. The present study aimed to investigate the potential pathological mechanism of the conversion from ADPC to CRPC by exploring the function of lung cancer metastasis-related protein 1 (LCMR1). We found that LCMR1 and glucocorticoid receptor α (GRα) were highly expressed in CRPC tissues, compared to ADPC tissues, and were accompanied by high concentrations of inflammatory factors. Knocking down LCMR1 or GRα in CRPC cells led to inhibition of metastasis and proliferation and induction of apoptosis. The expression of HSP90 and IL-6 was upregulated and that of androgen receptor was downregulated by knocking down LCMR1 or GRα in CRPC cells. Luciferase assay results indicated that the transcription of GRα was promoted by the LCMR1 promoter. The growth rate of CRPC cells in vivo was greatly decreased by knocking down LCMR1 or GRα. Lastly, CRPC cell sensitivity to enzalutamide treatment was found significantly enhanced by the knockdown of LCMR1. Taken together, LCMR1 might regulate the conversion of ADPC to CRPC by activating the GRα signaling pathway.

## Introduction

Prostate cancer is a common disease among the elderly. Globally, it is the second highest among malignant tumors for males [1,2]. In America, the morbidity of prostate cancer ranks first in cancers that endanger the health of males. Every year, approximately 680,000 new patients are diagnosed with prostate cancer and 220,000 patients died of prostate cancer [3]. Although the morbidity of prostate cancer in China is significantly lower than that in Western countries, it has maintained an upward trend in recent years [4].

In recent years, androgen deprivation therapy (ADT) has been considered as a therapeutic strategy for the treatment of advanced metastatic prostate cancer (ADPC), significantly delaying its progression [5,6]. However, 18–24 months after the treatment, the tumor types of almost all the patients transformed into castration-resistant prostate cancer (CRPC), which is the main cause of deaths in these patients [7]. Many factors are involved in the alteration from ADPC to CRPC. The abnormal activation of the androgen receptor (AR) signal pathway [8], activation of the P13K/PTEN/Akt/mTOR signaling pathway [9] and abnormal regulation of non-coding RNA were reported to play a role [10]. However, none of these processes can explain the pathogenesis of CRPC. Elucidation of the pathogenic mechanism of CRPC is critical for preventing and treating advanced prostate cancer.

Inflammation is an autoregulatory response to exogenous stimulation. However, various types of diseases, including the failure of organ function, are induced by uncontrolled inflammatory reactions, such as chronic cirrhosis and renal fibrosis [11]. Current reports indicate that approximately 20% of adult tumors are related to chronic inflammation [12]. The local microenvironment is activated by the relapse of inflammatory reactions, which results in the progression of apoptosis and necrosis to over proliferation in normal cells, accompanied by changes in genetics and epigenetics. As a result, malignant transformation and cancer formation occur in normal cells [13]. Kwon et al. reported that normal developmental and differentiation processes were destroyed by prostatitis in a mouse prostatitis model, which induced the malignant transformation of basal cells and the onset of prostate cancer [14]. It has been reported that the glucocorticoid receptor (GR) is closely related to the development and progression of inflammatory reactions [15]. Sawyers [16] reported that the potential growth ability without androgens could be achieved by prostate cancer by the activation of GR. Although the relationship between chronic inflammation and cancer has been widely and deeply investigated, the effects of chronic inflammatory reactions in CRPC have rarely been reported. In the present study, we speculated that LCMR1 is involved in the progression of ADPC to CRPC. Thus, we aimed to investigate the potential pathological mechanism of progression and provide a new target for the treatment of clinical CRPC.

## Methods and materials

Patients, specimens, and tumor cell isolation: Excisional tumor tissues were collected from 54 patients with ADPC and 54 patients with CRPC in the affiliated Xiaoshan Hospital，Hangzhou Normal University. All human experiments involved in this study were authorized by the ethical committee of the affiliated Xiaoshan Hospital，Hangzhou Normal University. The tissues were stored at 80°C for subsequent experiments. The connective tissues, adipocytes, and necrotic tissues within the tumor tissues were removed, followed by washing in Hanks buffer three times. Subsequently, the remaining tumor tissues were cut into pieces of 1–2 mm^3^. The pancreatic enzymes were used to digest the small pieces, and the serum was added to stop the reaction after 30 min of incubation. Cells were mixed by pipette repeatedly and filtered using a 70 μm cell strainer. The big, undigested pieces were removed, and the remaining filtered solution was centrifuged to remove the precipitate. The precipitate was resuspended in a DMEM cell culture medium containing 10% FBS and antibiotics. The cells were maintained at 37°C in a humidified chamber supplemented with 5% CO_2_. After passaging for 3–4 generations, the tumor cells were successfully isolated.

Immunohistochemistry: The tissues were placed into a plate filled with pre-cooled normal saline. They were later embedded in paraffin, sectioned, and incubated with the LCMR1 antibody (Bioss, 1:1,000). After incubating overnight at 4°C, the slides were incubated with HRP polymer and visualized under an optical microscope (Olympus) [17].

Real-time RT-PCR: Total RNA was extracted from the tissues using an RNA Extraction Kit (Takara) according to the manufacturer’s instructions. The extracted RNA was quantified using a NanoDrop spectrophotometer (NanoDrop Technologies). A specific RT primer was used to reverse transcribe complementary DNA. SYBR Premix Ex TaqTM (Takara) with an Applied Bio-Rad CFX96 Sequence Detection System (Applied Biosystems) was used for real-time PCR. The expression levels of LCMR1 and GRα were determined by the threshold cycle (Ct), and relative expression levels were calculated by the 2^−ΔΔCt^ method after normalization with the expression of U6 small nuclear RNA. GAPDH expression in the tissue was used as a negative control. Three independent assays were performed. The information on the primers is shown in Table 1 [18].

**Table 1.** The sequences of primers for LCMR1, GRα and GAPDH

primer name	primer sequence (5ʹ-3ʹ)	primer length (bp)
LCMR1 forward	AACAGAGCCGTACCCAGGAT	20
LCMR1 reverse	GGGTGGTCTGGACATTGTC	19
GRα forward	AACTGGCAGCGGTTTTATCAA	21
GRα reverse	TGGAAGCAATAGT TAAGGAGATTTT	26
GAPDH forward	CAATGACCCCTTCATTGACC	20
GAPDH reverse	GAGAAGCTTCCCGTTCTCAG	20

### Transfection

CRPC cells were seeded in a 6-well culture plate 24 h before transplantation. LCMR1 or GRα siRNAs were transfected using Lipofectamine 2000 (Invitrogen) and X-treme GENE HP DNA Transfection Reagent (Invitrogen) according to the manufacturer’s instructions. CRPC cells were collected 24 or 48 h later and subjected to further analysis. The assays were performed in triplicates, and more than nine wells were treated with the same type of siRNA.

Wound healing assay [19]: The cells were grown to confluence and a linear wound was made by scraping a non-opening Pasteur pipette across the confluent cell layer, 24 h after treatment with mitomycin C (10 μg/mL). The cells were washed twice to remove the detached cells and debris. Then, the size of the wounds was observed and measured at the indicated times.

MTT assay for assessing cell growth [20]. Cell viability was detected using the semi-automatic 3-(4,5-dimethyl-thiazol-2-yl)-2,5- diphenyltetrazolium bromide (MTT, Sigma) assay according to the manufacturer’s instructions. Briefly, the cells were seeded in a 96-well plate and incubated overnight. MTT (0.5 mg/mL) was added to the medium and the cells were incubated for 4 h. The formazan precipitate was dissolved in approximately 200 μL of dimethyl sulfoxide (DMSO), and the absorbance at 490 nm was measured using a benchmark microplate reader (Bio-Rad, CA). Three independent assays were performed.

Luciferase Assays [21]: Cells were plated at 5 × 10^4^ cells per well in 24-well plates. The next day, 200 ng pMIR-REPORT Luciferase vector, including the 3′ untranslated region (UTR) of GRα (with the WT or mutant LCMR1 promoter response element) and blank vector, were transfected using Lipofectamine 2000 (Invitrogen). Luciferase assays were performed using the dual-luciferase reporter assay system (Promega) 48 h after transfection.

Flow cytometry for testing the apoptosis of cells [22]: The cells were collected into 1.5 mL tubes. Each tube was added with 10 μL fluorescently-labeled Annexin V reagent and 5 μL PI reagent were added to each tube. Each tube was incubated for 10 min at room temperature. Approximately 200 μL of cells were added into the flow tube containing 2 mL of PBS and tested by flow cytometry (BD). Three independent assays were performed.

Detection of inflammatory factors by ELISA [23]: The cells were centrifuged at 1,000 × g for 5–10 min. The supernatant and tumor homogenate was used to detect interleukin-1β (IL-1β), interleukin-6 (IL-6), and tumor necrosis factor-α (TNF-α) using ELISA kits (eBioscience) according to the manufacturer’s instructions.

Western blot assay [24]: Total proteins were isolated from tissues or cells using the Nuclear and Cytoplasmic Protein Extraction Kit (Beyotime, China). Approximately 40 μg of protein was separated on a 12% SDS-polyacrylamide gel (SDS-PAGE), and the gel was transferred to a polyvinylidene difluoride (PVDF) membrane(Millipore, Massachusetts, USA). The membrane was blocked with 5% nonfat dry milk in TBST (Tris-buffered saline/0.1% Tween-20, pH 7.4) for 1 h at room temperature and incubated overnight with primary rabbit anti-human antibodies against LCMR1 (1:1,000), GRα (1:1,000), HSP90 (1:1,000), IL-6 (1:1,000), and AR (1:1,000) (Abcam, USA). A horseradish peroxidase-conjugated antibody against rabbit IgG (1:5,000, Abcam, USA) was used as the secondary antibody. Blots were incubated with ECL reagents (Beyetime, Jiangsu Province, China) and exposed to Tanon 5200-multi to detect protein expression. Three independent assays were performed.

Xenograft experiment [25].: Twelve BABL/c nude mice were purchased from the Beijing Vital River Laboratory Animal Technology Co., Ltd. The animals were randomly divided into four groups. 1) ADPC where animals were subcutaneously injected with normal ADPC cells. 2) GRα knockdown (KD) CRPC with GRα-KD CRPC cells. 3) LCMR1 KD CRPC with LCMR1 KD CRPC cells and 4) CRPC with CRPC cells. In the xenograft experiment, the planted GRα KD CRPC cells were established by transfecting CRPC cells with a lentivirus vector (Thermo Fisher, Massachusetts, USA) containing siRNA against GRα. The planted LCMR1 KD CRPC cells were constructed by transfecting CRPC cells with a lentivirus vector (Thermo Fisher, Massachusetts, USA) containing the siRNA against LCMR1. The length (L) and width (W) of the tumor were measured and recorded every two days after the cells were injected. The volume of the tumor (V) was calculated using the formula: V = L × W^2^ × 0.5. The data were recorded for 16 days after the cells were injected. Subsequently, the animals were sacrificed with CO_2_, and the tumors were collected and weighed.

### Statistical analysis

Statistically significant differences for continuous variables were determined using one-way analysis of variance (ANOVA) with the least significant difference (LSD) test for normally distributed data. All tests were performed using the GraphPad Prism 5 software. Differences were considered significant when the P value was less than 0.05.

### Ethics statements

All animal experiments involved in this study were authorized by the ethical committee of affiliated Xiaoshan Hospital，Hangzhou Normal University and carried out according to the guidelines for care and use of laboratory animals and to the principles of laboratory animal care and protection.

## Results

The present study aimed to investigate the potential pathological mechanism of the progression of ADPC into CRPC by investigating the function of LCMR1. Firstly, the relative expression levels of LCMR1 and GRα in clinical ADPC and CRPC tissues were detected and compared. Then, the metastasis, proliferation, and apoptosis, as well as the state of AR signaling in LCMR1 knockdown CRPC cells were investigated. Subsequently, GRα was knocked down in CRPC cells, followed by detection of metastasis, proliferation, and apoptosis, accompanied by evaluation of the activity of the AR signaling pathway. The correlation between LCMR1 and GRα was assessed using a luciferase reporter assay. Lastly, the involvement of LCMR1 in the development of CRPC was confirmed using a xenograft experiment and an in vitro enzalutamide sensitivity assay.

**Figure 1.**
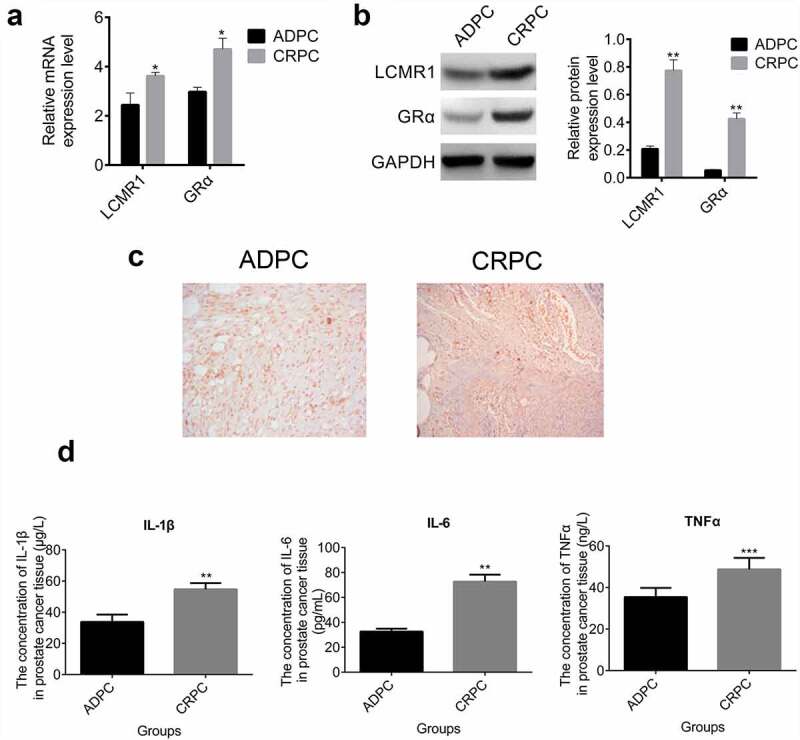
(a). The expression of LCMR1 and GRα at the mRNA level in ADPC and CRPC tissues was detected by qRT-PCR. (b). The protein expression levels of LCMR1 and GRα in ADPC and CRPC tissues were determined by Western blotting. (c). The expression of LCMR1 in ADPC and CRPC tissues was evaluated by immunohistochemistry. (d). The concentration of IL-1β, IL-6, and TNF-α was determined by ELISA in ADPC and CRPC tissues. Three independent assays were performed. *P < 0.05 vs. ADPC, **P < 0.01 vs. ADPC, ***P < 0.001 vs. ADPC.

LCMR1 and GRα were highly expressed in clinical CRPC tissue: To determine the expression levels of LCMR1 and GRα in the ADPC and CRPC tissues, excisional tumor tissues were collected and qRT-PCR, Western blotting, and immunohistochemistry were performed. As shown in Figure 1a, both LCMR1 and GRα were expressed at higher levels in clinical CRPC tissues than in ADPC tissues (*P < 0.05). Figure 1b shows that at the protein level, compared with the ADPC group, both LCMR1 and GRα were highly expressed in the CRPC group (**P < 0.01). The immunohistochemistry results are shown in Figure 1c, which indicated that the expression level of LCMR1 in CRPC tissues was higher than that in ADPC tissues. Figure 1d shows that the concentration of IL-1β, IL-6, and TNF-α in CRPC tissues was significantly higher than that in ADPC tissues (**P < 0.01,***P < 0.001). We hypothesized that the overexpression of LCMR1 in CRPC tissues might be related to inflammation activation.

**Figure 2.**
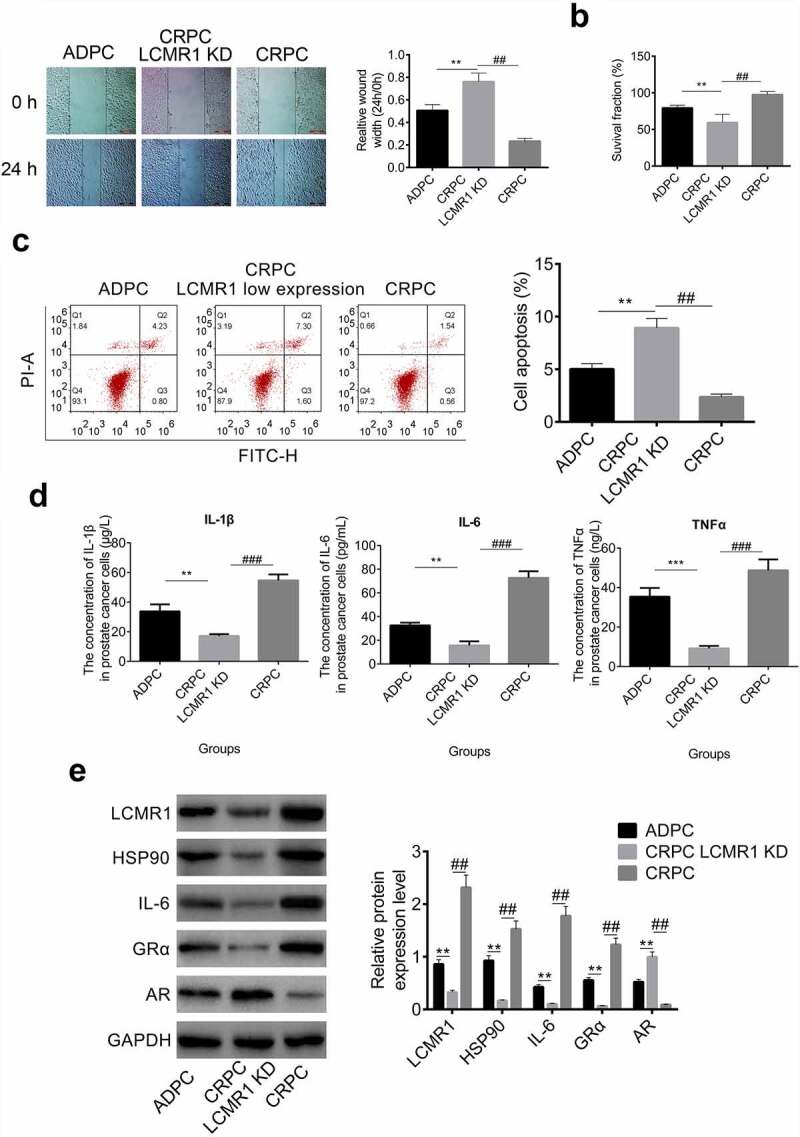
(a). The metastasis of prostate cancer cells was evaluated by wound healing study at 0 and 24 h. (b). The proliferation of prostate cancer cells was determined by MTT assay. (c). The apoptotic rate of prostate cancer cells was determined by flow cytometry. (d). The concentration of IL-1β, IL-6, and TNF-α was determined by ELISA in the supernatant of prostate cancer cells. (e). The protein expression levels of LCMR1, GRα, HSP90, IL-6, and AR were determined by Western blotting. **P < 0.01 vs. ADPC, ***P < 0.001 vs. CRPC, ##P < 0.01 vs. CRPC, ###P < 0.001 vs. CRPC.

The function of LCMR1 in the metastasis, proliferation, and apoptosis of CRPC cells: LCMR1 was found to be highly expressed in CRPC cells compared with ADPC cells. LCMR1 KD CRPC cells were established with LCMR1 siRNAs to evaluate their function. As shown in Figure 2a, in the wound healing study, the relative wound width at 24 h versus 0 h was greatly increased in LCMR1 KD CRPC cells, compared to that in CRPC cells (##P < 0.01) and in ADPC cells (**P < 0.01). These results indicate that CRPC metastasis was significantly inhibited after LCMR1 knockdown. To evaluate the effects of LCMR1 on proliferation ability, an MTT assay was performed. As shown in Figure 2b, the survival fraction of LCMR1 KD CRPC cells was significantly lower than that of both CRPC cells (##P < 0.01) and ADPC cells (**P < 0.01). The knockdown of LCMR1 exerted an inhibitory effect on the proliferation of CRPC cells. Apoptosis results are shown in Figure 2c. The apoptotic rates of ADPC, LCMR1 KD CRPC, and CRPC cells were 5.03%, 8.90%, and 2.10%, respectively. The knockdown of LCMR1 significantly promoted the apoptotic rate of CRPC cells. Inflammatory factors were detected in the cell culture supernatant. As shown in Figure 2d, the concentrations of IL-1β, IL-6, and TNF-α in the supernatant of LCMR1 KD CRPC cells was significantly lower than that in ADPC cells (**P < 0.01, ***P < 0.001) and CRPC cells (###P < 0.001).

To further investigate the impact of LCMR1 on the GRα and AR signaling pathways, the expression levels of HSP90, IL-6, GRα, and AR were determined by Western blotting. The protein bands and gray values are shown in Figure 2e. First, after LCMR1 knockdown, the protein expression level was greatly inhibited, compared to both CRPC cells (##P < 0.01) and ADPC cells (**P < 0.01), which indicated that the expression of LCMR1 was successfully decreased by the siRNA. After knocking down LCMR1, the expression of GRα was also greatly inhibited, as was the expression of the downstream proteins (HSP90 and IL-6) of the GRα signal pathway (##P < 0.01). However, AR was highly expressed in LCMR1 KD CRPC cells, compared to both CRPC cells (##P < 0.01) and ADPC cells (**P < 0.01). These data indicate that the GRα signaling pathway is inhibited and the AR signaling pathway is activated by knocking down the expression of LCMR1 in CRPC cells.

**Figure 3.**
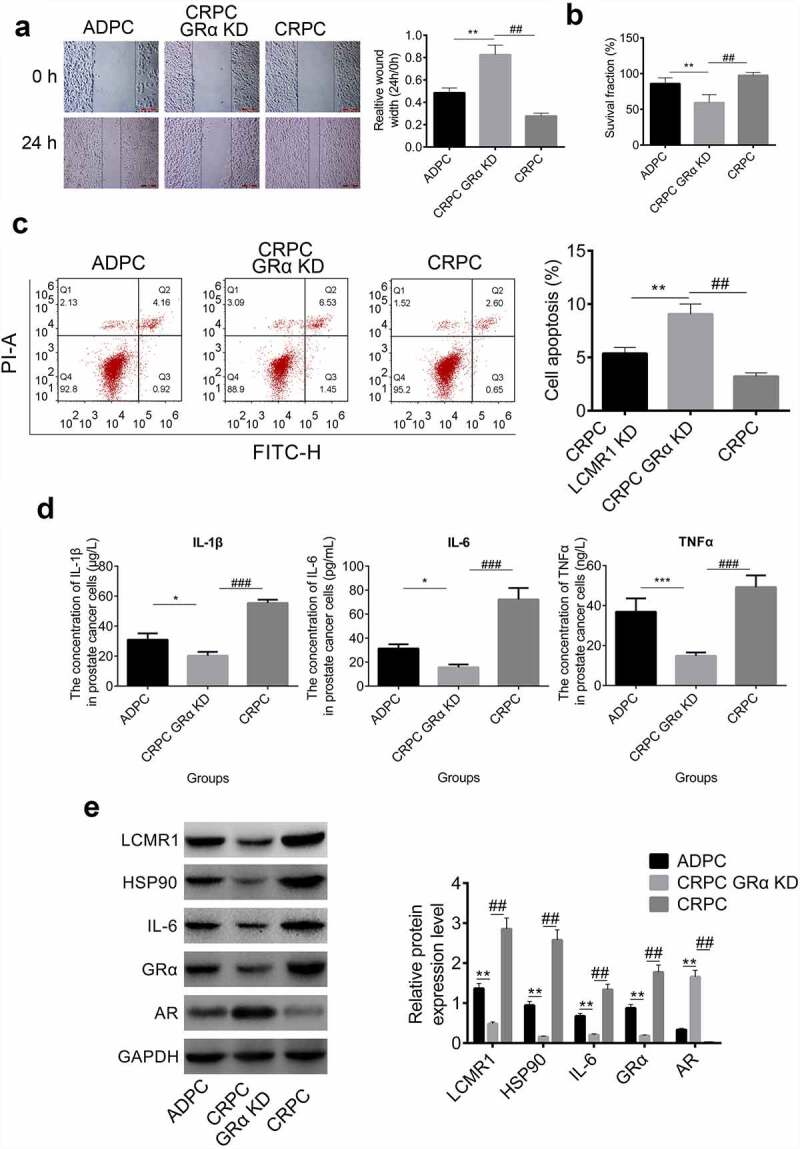
(a). The ability of metastasis of prostate cancer cells was evaluated by wound healing study at 0 h and 24 h. (b). The proliferation of prostate cancer cells was detected by MTT assay. (c). The apoptotic rate of prostate cancer cells was determined by flow cytometry. (d). The concentration of IL-1β, IL-6, and TNF-α was determined by ELISA in the supernatant of prostate cancer cells. (e). The protein expression levels of LCMR1, GRα, HSP90, IL-6, and AR were determined by Western blotting. *P < 0.05 vs. ADPC, **P < 0.01 vs. ADPC, ***P < 0.001 vs. CRPC, ##P < 0.01 vs. CRPC, ###P < 0.001 vs. CRPC.

The function of GRα in the metastasis, proliferation, and apoptosis of CRPC cells: GRα was also found to be highly expressed in CRPC cells compared to ADPC cells, GRα KD CRPC cells were established with GRα siRNAs to evaluate its function. As shown in Figure 3a, in the wound healing study, the relative wound width at 24 h versus 0 h was greatly promoted in GRα KD CRPC cells, compared with that in CRPC cells (##P < 0.01). Compared with ADPC cells, the relative wound width at 24 h versus 0 h was higher in GRα KD CRPC cells (**P < 0.01). These results indicate that CRPC metastasis was significantly inhibited after GRα knockdown. To evaluate the effects of GRα on the proliferation ability of CRPC cells, an MTT assay was performed. As shown in Figure 3b, the survival fraction of GRα KD CRPC cells was significantly lower than that of both CRPC cells (##P < 0.01) and ADPC cells (**P < 0.01). GRα KD exerted an inhibitory effect on the proliferation of CRPC cells. Apoptosis results are shown in Figure 3c. The apoptotic rates of ADPC, GRα KD CRPC, and CRPC cells were 5.08%, 7.89%, and 3.25%, respectively. GRα KD significantly increased the apoptotic rate of CRPC cells. Inflammatory factors were detected in the cell culture supernatant. As shown in Figure 3d, the concentrations of IL-1β, IL-6, and TNF-α in the supernatant of GRα KD CRPC cells was significantly lower than that of ADPC cells (**P < 0.01, ***P < 0.001) and CRPC cells (###P < 0.001).

To further investigate the impact of GRα on the LCMR1 expression level and AR signaling pathway, a Western blot assay was performed. The protein bands and gray values are shown in Figure 3e. First, after knocking down GRα, the protein expression level was greatly inhibited, compared to both CRPC (##P < 0.01) and ADPC cells (**P < 0.01), which indicated that the expression of GRα was successfully decreased by the siRNA. The expression levels of the downstream proteins (HSP90 and IL-6) of the GRα signaling pathway were decreased by GRα KD (##P < 0.01 vs. CRPC) and LCMR1 was also downregulated (##P < 0.01 vs. CRPC), which indicated that a two-way regulation mechanism was involved between LCMR1 and GRα. However, AR was highly expressed in GRα KD CRPC cells, compared to both CRPC (##P < 0.01) and ADPC cells (**P < 0.01).

**Figure 4.**
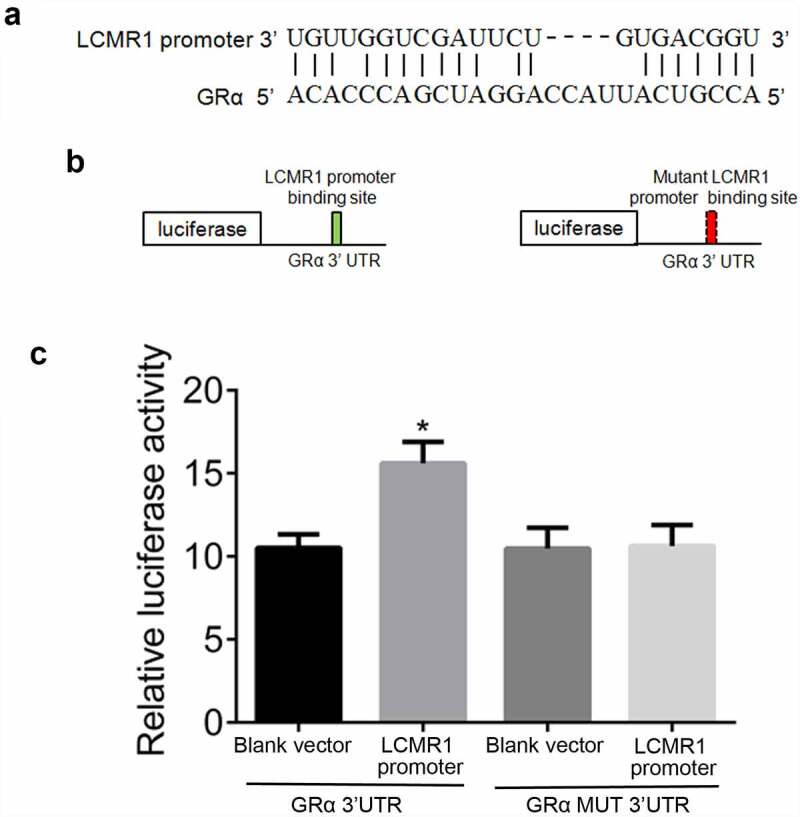
(a). LCMR1 promoter and the LCMR1 promoter-binding site in the 3′ UTR of GRα. (b). Design of an LCMR1 promoter reporter vector containing a CMV promoter driving expression of a luciferase cDNA fused to the GRα 3′ UTR or a mutated GRα 3′ UTR. (c). The 3′ UTR of GRα mediates LCMR1 promoter control of GRα expression. 293 T cells were transfected with a reporter vector consisting of a luciferase cDNA fused to the 3′ UTR of GRα which contains a binding site of LCMR1 promoter. Another vector contained the luciferase cDNA fused to a GRα 3′ UTR with a mutant LCMR1 promoter-binding site.

LCMR1 might be the transcription factor for GRα: To further explore the relationship between LCMR1 and GRα, a luciferase assay was performed. Computational analysis revealed a potential binding site for the LCMR1 promoter within the 3′ UTR of GRα was found (Figure 4a). To test the idea that the LCMR1 promoter induced the transcription of GRα through this site, we constructed a reporter vector consisting of a luciferase cDNA followed by the 3′ UTR of GRα (Figure 4b). A luciferase reporter vector fused to the GRα 3′ UTR but with a mutant LCMR1 promoter response element was constructed. The luciferase reporter vector with a WT or mutant LCMR1 promoter response element was transfected into HEK293T cells. The LCMR1 promoter increased luciferase activity of the reporter vector containing the LCMR1 promoter response element (Figure 4c) (*P < 0.05, vs. blank vector). These results suggest that the LCMR1 promoter induces the transcription of GRα by acting on a response element in the 3′ UTR of GRα.

**Figure 5.**
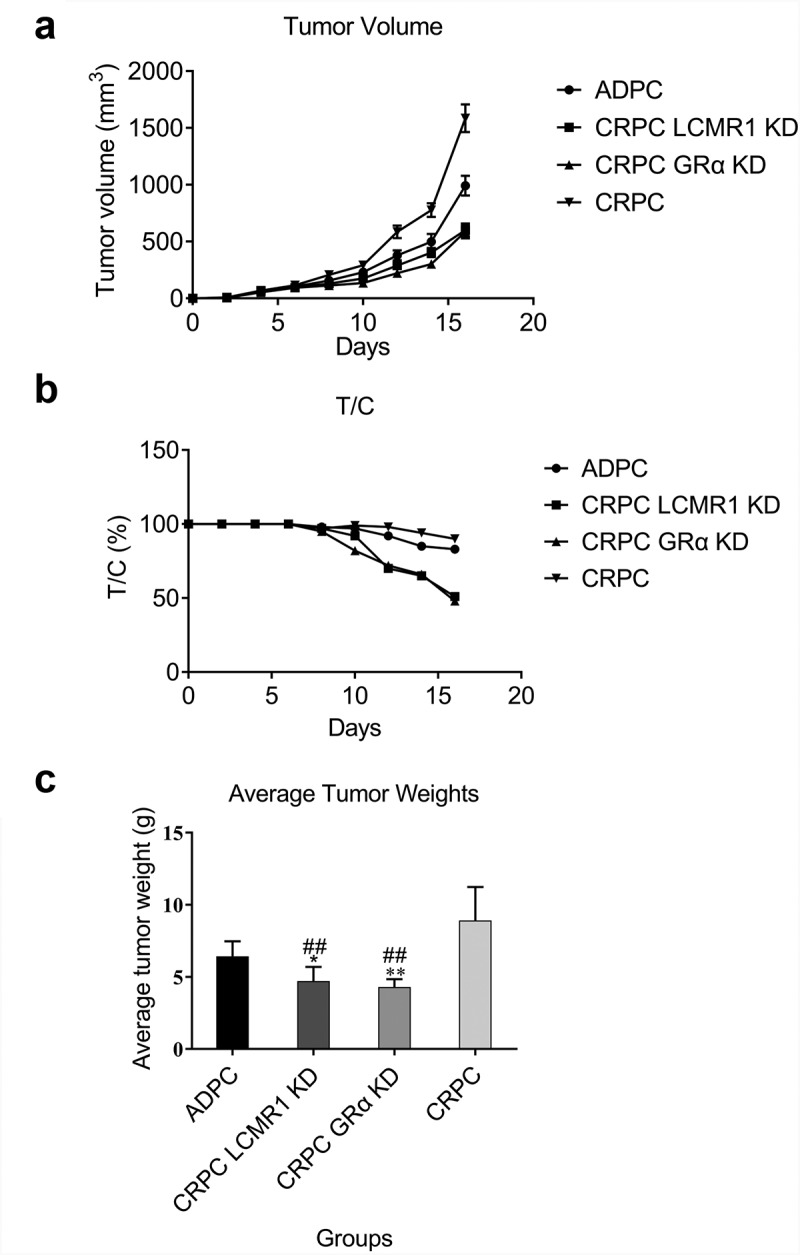
The xenograft model was established by injecting with different prostate cancer cells. (a). Curves of tumor volume versus administration time; (b). Curves of T/C versus administration time. (c). Average tumor weight in mice. *P < 0.05 vs. ADPC, **P < 0.01 vs. ADPC, ##P < 0.01 vs. CRPC.

The function of LCMR1 and GRα in tumor growth in a xenograft model: To further investigate the effects of LCMR1 and GRα on tumor growth in vivo, a xenograft model was established in nude Babl/c mice. As shown in Figure 5a, the growth rate of tumors in the LCMR1 KD CRPC group was significantly lower than that in both the ADPC and CRPC groups. The same data are shown in the GRα KD CRPC group. Figure 5b shows that ten days after the injection of tumor cells, the T/C decreased greatly in both the LCMR1 KD CRPC group and GRα KD CRPC group, compared to the ADPC and CRPC groups. The final average tumor weights of mice from the LCMR1 KD CRPC group and GRα KD CRPC group were significantly lesser than those in the ADPC (*P < 0.05, **P < 0.01) and CRPC groups (##P < 0.01). These data indicate that tumor growth was significantly inhibited by knocking down LCMR1 and GRα in CRPC cells.

**Figure 6.**
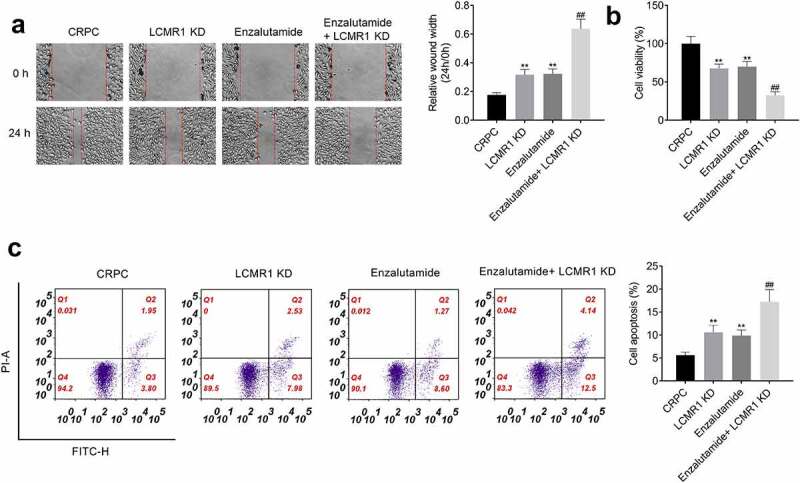
The CRPC cell sensitivity to enzalutamide treatment was enhanced by the knockdown of LCMR1. (a). The metastasis of prostate cancer cells was evaluated by wound healing study at 0 and 24 h. (b). The proliferation of prostate cancer cells was determined by MTT assay. (c). The apoptotic rate of prostate cancer cells was determined by flow cytometry. **P < 0.01 vs. CRPC, ##P < 0.01 vs. Enzalutamide.

Knockdown of LCMR1 promoted CRPC cell sensitivity to enzalutamide treatment: To further confirm that LCMR1 was involved in the LCMR1 is involved in the transforming process from ADPC to CRPC, CRPC cells or LCMR1-knockdow CRPC cells were treated with enzalutamide, respectively. Four groups were divided: CRPC (CRPC cells were treated with blank medium), LCMR1 KD (LCMR1-knockdow CRPC cells were treated with blank medium), Enzalutamide (CRPC cells were treated with 20 μM enzalutamide) [26], and Enzalutamide+ LCMR1 KD (LCMR1-knockdow CRPC cells were treated with 20 μM enzalutamide). As shown in Figure 6a, in the wound healing study, compared to the CRPC group, the relative wound width at 24 h versus 0 h in both the LCMR1 KD group and Enzalutamide group was significantly promoted. Compared to the Enzalutamide group, the relative wound width at 24 h versus 0 h in the Enzalutamide+ LCMR1 KD group was further elevated. Additionally, significantly declined cell viability was observed in the LCMR1 KD group and Enzalutamide group, compared to the CRPC group. However, the cell viability in Enzalutamide treated LCMR1 knockdown CRPC cells was significantly reduced compared to the Enzalutamide group. Lastly, the apoptotic rate in the LCMR1 KD group and Enzalutamide group was significantly increased from 5.6% to 10.6% and 9.9%, respectively. However, compared to the Enzalutamide group, the apoptotic rate in the Enzalutamide+ LCMR1 KD group was dramatically elevated from 9.9% to 17.3% (**P < 0.01 vs. CRPC, ##P < 0.01 vs. Enzalutamide). These data suggested that the CRPC cell sensitivity to enzalutamide treatment was enhanced by the knockdown of LCMR1.

In the present study, the mechanism underlying the conversion from ADPC to CRPC will be investigated, which may be beneficial to bring new insights into the treatment of clinical advanced prostate cancer.

## Discussion

The development of ADPC into CRPC during the treatment of advanced metastatic prostate cancer with ADT majorly leads to death in patients [27–29]. Multiple factors have been reported to be involved in tumor type changes. The lncRNA CCAT1 was reported to be an oncogenic factor in the progression of CRPC with different regulatory mechanisms in the nucleus and cytoplasm of cells [30]. Guan H [31] reported that the BRD4/AR signaling pathway was significantly activated when progressing from ADPC to CRPC, and miR-200a could suppress prostate cancer progression by inhibiting this signaling pathway. In the present study, LCMR1 was found to be highly expressed in the excised tumor tissues from CRPC patients, compared to that in ADPC patients. We hypothesized that LCMR1 may play an important role in the progression of ADPC to CRPC.

LCMR1 is the main component of the mediator complex which regulates the transcription of downstream genes by combining DNA [32]. It was reported that LCMR1 was involved in promoting the progression of lung cancer, osteosarcoma, and bowel cancer [33]. Although our previous work showed that the regulatory mechanism between LCMR1 and AR is important for the development of ADPC, the function and role of LCMR1 in the formation of CRPC remain unknown. A PCR array was used in our previous work, and we found that LCMR could regulate the expression of GRα and its downstream proteins, indicating that LCMR1 could regulate the GRα signaling pathway to impact cell functions. In addition, in the ADPC cell line (LNCaP), AR was highly expressed and GRα was poorly expressed. However, in the CRPC cell line (DU145), the expression levels of AR and GRα were the opposite. In the present study, ADPC and CRPC cells were isolated from the excised tumor tissues of patients with ADPC and CRPC, respectively. After LCMR1 was knocked down in the CRPC cells, the cell metastasis and proliferation ability of CRPC cells were significantly inhibited and cell apoptosis was induced, which indicated that LCMR1 could promote the progression of CRPC. In addition, the expression of AR was promoted and the GRα signaling pathway was inhibited, which was consistent with the findings in LNCaP and DU145 cells. The sensitivity of enzalutamide is regarded as a distinguishing method for ADPC and CRPC, which is previously used to confirm the regulatory function of MLL5α in the transformation from ADPC to CRPC [26]. In the present study, we found that the sensitivity of CRPC cells to enzalutamide was enhanced by the knockdown of LCMR1, which further verified that LCMR1 was involved in the development of CRPC. Based on these results, we hypothesized that LCMR1 might promote the progression of CRPC by regulating the transcription of GRα to activate its downstream signaling pathway.

The functions of GR are extensive and complex, and it is involved in multiple physiological processes, including growth, energy metabolism, the immune system, and the cardiovascular system. There are two main subtypes of GR: GRα and GRβ. GRα regulates the progress of transcription by combining with glucocorticoid (GC). However, GRβ could not combine with GC and competitively inhibited the function of GRα [34]. It has been reported that the introduction of GC inhibits the synthesis of AR and ameliorates the symptoms of prostate cancer. Sawyers reported that GR could be activated by GC, which induced the growth of prostate tumor cells via an androgen-independent mechanism. In this case, the patients were resistant to antiandrogenic drugs. However, the introduction of a GRα antagonist could promote the increased sensitivity of prostate cancer to antiandrogenic drugs [16]. In the present study, GRα expression was downregulated by LCMR1 knockdown in CRPC cells. The ability of CRPC cells to metastasize and proliferate was significantly inhibited and apoptosis was greatly induced by knocking down the expression of GRα in CRPC cells. The in vivo study also showed that the growth rate of CRPC tissue in nude mice decreased greatly after knocking down the expression of GRα in CRPC cells. The luciferase reporter assay indicated that the LCMR1 promoter could promote the transcription of the GRα 3′ UTR. These data, together with the investigation of LCMR1 in CRPC, indicated that the transfer from ADPC to CRPC might be related to the activation of the GRα signaling pathway, which was induced by the overexpression of LCMR1.

The expression of inflammatory factors was investigated to explore the effects of LCMR1 overexpression in CRPC. The expression of LCMR1 was relatively higher in the CRPC tissue than in the ADPC tissue, accompanied by a high concentration of inflammatory factors. We assume that the upregulation of LCMR1 might be caused by chronic inflammation. Interestingly, after knocking down LCMR1 or GRα in CRPC cells, the release of inflammatory factors was also inhibited. These data might be explained by the inflammation-activating ability of GRα [15]. However, further investigation should be performed to verify whether chronic inflammation is the main factor that results in the over-expression of LCMR1 and whether there was a malignant circulation in the ‘inflammation-LCMR1-GRα-inflammation’ axis.

## Conclusion

LCMR1 enables the progression of ADPC into CRPC by activating the GRα signaling pathway, accompanied by the activation of inflammation.

## Highlights

LCMR1 and GRα were found highly expressed in the CRPC tissues.

A high concentration of inflammatory factors was observed in CRPC tissues.

LCMR1 regulates the conversion of ADPC to CRPC by activating the GRα signal pathway.
